# Age assurance for online sexual content: applying the health policy triangle to UK early signals and transferable safeguards for sub-Saharan Africa

**DOI:** 10.3389/fpubh.2026.1762704

**Published:** 2026-04-16

**Authors:** Moses Ifeatu Nwuzoh, Stanley Chinedu Eneh, Collins Chibueze Anokwuru, Onyeka Chukwudalu Ekwebene, Moses Obinna John, Runyi Bassey Ogbet, Ruth Senibi, Samson Adiaetok Udoewah

**Affiliations:** 1 Department of Food Science and Technology, Chukwuemeka Odumegwu Ojukwu University, Igbariam, Anambra, Nigeria; 2 Youth in Research Hub, Enugu, Nigeria; 3 Department of Public Health, Sheffield Hallam University, Sheffield, United Kingdom; 4 Department of Community Health, Obafemi Awolowo University, Ile-Ife, Osun, Nigeria; 5 IVAN Research Institute, Enugu, Nigeria; 6 Department of Public Health, Federal University of Technology Owerri, Owerri, Nigeria; 7 Department of Biostatistics and Epidemiology, East Tennessee State University, Johnson City, TN, United States; 8 Public Administration/Policy, LIGS University, Honolulu, HI, United States; 9 International Foundation Against Infectious Diseases in Nigeria, Abuja, Nigeria; 10 Department of Public Health, Monroe University, New York, NY, United States; 11 Department of Public Health Nursing, Africa Centre of Excellence in Public Health and Toxicological Research, University of Port Harcourt, Port Harcourt, Nigeria

**Keywords:** age assurance, child online protection, equity, health policy triangle, sub-Saharan Africa, United Kingdom online safety act, digital governance, online safety regulation

## Abstract

Children’s exposure to sexually explicit online material has been associated with early sexualisation, distorted gender norms, risky sexual behaviours, and adverse mental health outcomes, with first exposure often occurring before the age of 12 and increasingly through mainstream digital platforms. Age assurance has therefore emerged as a regulatory approach that shifts the responsibility of restricting underage access from parents to digital services. The United Kingdom Online Safety Act 2023 establishes one of the first comprehensive statutory age assurance frameworks, combining risk-based duties, regulatory guidance, multiple verification pathways, privacy protections, and staged enforcement. This study applies a desk-based policy analysis, guided by the Health Policy Triangle, to examine the regulatory architecture of the United Kingdom age assurance framework and assess the conditional transferability of its safeguards to sub-Saharan Africa. The sources included legislation, regulatory guidance, official policy documents, civil society analyses, peer-reviewed literature, and early implementation reports published between 2020 and 2025. Early implementation signals indicate platform compliance actions, reported traffic reductions to certain adult sites, increased circumvention behaviour, including the use of virtual private networks, and mixed public responses characterised by broad support for child protection alongside concerns about the effectiveness of the measures and the sharing of identity credentials. These indicators are interpreted as preliminary governance and implementation signals rather than the evidence of policy effectiveness. Five policy design elements appear potentially adaptable under equity and capacity constraints: risk-based proportionality, multiple and document-free verification pathways, transparency and vendor accountability, staged supervision and enforcement, and cross-regulator coordination. The study provides a policy analytic framework for country-level adaptation in sub-Saharan Africa and highlights priorities for context-sensitive implementation and future evaluation.

## Introduction

Children’s exposure to sexually explicit online material is linked to early sexualisation, unrealistic gender norms, risky sexual behaviours, and adverse mental health outcomes, with evidence suggesting that initial exposure often occurs before the age of 12, sometimes even in later stages of childhood (1–3). Reports show that adolescents’ exposure to sexually explicit material often on mainstream platforms (social media, search engines, and video-sharing services) rather than on dedicated sites (pornography or adult content sites), prompting concerns about how easily under-18 users can access adult content (3, 4).

In response to these risks, age assurance has gained prominence as a policy mechanism that shifts the responsibility of preventing underage access from parents to digital platforms and service providers (5). Age assurance methods assess whether a user is likely to be a child or an adult without necessarily confirming the exact age (5). These methods include verification approaches (such as government identification and payment instruments) and estimation approaches (such as AI-based facial or voice models and non-biometric behavioural signals) (5, 6).

The United Kingdom (UK) is one of the first countries to legislate comprehensive age assurance duties at national scale through the Online Safety Act 2023 (OSA) (7). The Act requires services to apply risk-based and proportionate measures, with the Office of Communications (Ofcom) empowered to issue guidance, conduct investigations, and apply sanctions (7–10). Supporters argue that the Act strengthens child protection in digital environments, while critics warn of risks relating to privacy, data protection, exclusion of adults without credentials, and potential algorithmic bias associated with artificial intelligence systems (11–13).

By contrast, sub-Saharan Africa (SSA) presents a complex context for transferring such regulatory models. The region has experienced rapid growth in Internet access, with International Telecommunication Union data indicating that Internet use in Africa increased from approximately 16% of the population in 2015 to over 37% by 2023, with youth adoption rates typically higher than national averages (14). A multi-country Demographic and Health Survey (DHS) analysis covering nine SSA countries (Burkina Faso, Côte d’Ivoire, Ghana, Kenya, Lesotho, Madagascar, Mozambique, Rwanda, and Tanzania), including adolescents aged 15 years and above and adult women, provides quantitative evidence that links Internet use with sexual risk behaviours such as multiple partnerships and non-condom use (15). Furthermore, evidence from Nigeria reveals that 83% of adolescents were regular social media users, of whom 47% had engaged in at least one risky sexual behaviour (16).

At the same time, the region continues to face uneven regulatory frameworks, limited identification coverage, and widespread shared device access (17–20). In these conditions, rigid single-route verification models may unintentionally increase digital exclusion, particularly where identification gaps, uneven regulatory capacity, and variation in parental mediation and supervision of children’s Internet use, especially outside formal school settings, remain common (18, 20, 21).

Taken together, regional exposure patterns, access conditions, and governance constraints indicate that any transferable regulatory model for SSA must combine child protection safeguards with design flexibility, proportionality, and multiple compliance pathways. In this study, the UK age assurance framework serves as the primary reference model because it integrates statutory platform duties, regulator-issued technical guidance, risk-based assurance standards, and staged enforcement within a single operational structure (7–10). Comparable regimes are more limited in scope. The European Union Digital Services Act requires platforms to apply appropriate and proportionate measures to protect minors, including possible age assessment tools, but it does not establish a detailed statutory age assurance regime equivalent to the UK duties (22). Australia’s Online Safety Act centres on complaint handling and removal powers (23), while the United States Children’s Online Privacy Protection Act focuses on personal data protection for younger children rather than platform-wide assurance duties (24). The UK framework, therefore, provides a useful analytical reference for examining adaptation in equity-constrained and capacity-variable contexts.

While child online safety is widely discussed in legal, technological, and digital rights scholarship (5, 6, 25), the majority of work focuses on normative, technical, or rights-based issues rather than structured policy design and cross-context transfer. There is a scarcity of studies applying formal policy analysis frameworks to examine how age assurance regulations are developed, implemented, and adapted across regulatory environments (3, 5, 6). As a result, links between policy context, actors, process, and content remain under-analysed, leaving a gap between normative debate and operational policy design.

Building on this gap and the SSA context, this study applies a structured desk-based policy analysis using the Health Policy Triangle framework to analyse the UK age assurance regulatory intervention and assess how policy context, actors, process, and content shape feasible safeguards (7–10, 26), particularly where post-implementation evaluation remains limited. This framing links online protection measures to public health, equity, and governance considerations and supports operational policy design rather than normative discussion alone.

Guided by this framework, the review aims to: (1) analyse the UK age assurance policy framework (AAPF) using the Health Policy Triangle; (2) identify early non-causal implementation signals; and (3) assess which policy design elements are transferable to SSA contexts, with attention to equity and feasibility.

### Desk-based policy analysis approach

This study employed a desk-based policy analysis approach, following established methods in health policy and systems research, in which regulatory texts, policy documents, and institutional guidelines are examined using structured analytical frameworks rather than primary data collection or causal effect estimation (26–29). Documentary analysis is commonly used to examine regulatory architecture, governance arrangements, and policy transfer conditions where experimental or primary data are unavailable, particularly in emerging policy areas and low- and middle-income settings (26–28, 30).

Policy sources relating to the UK AAPF were identified through purposive searches of government and regulator websites, legal and policy databases, and citation tracing from key regulatory and academic references. Materials included legislation, regulatory guidance, official policy documents, parliamentary materials, civil society analyses, peer-reviewed literature, and early implementation reporting. Documents published between 2020 and 2025 were prioritised to capture the policy development and early implementation period following the enactment of the Online Safety Act. Table 1 summarises the principal regulatory and policy documents reviewed.

**Table 1:** Key policy and regulatory documents were reviewed in the desk-based analysis of the United Kingdom age assurance policy framework.

Documents or source	Type of document	Main focus of the document
United Kingdom Online Safety Act 2023 (7)	Primary legislation	Establishes statutory duties requiring platforms to protect children online, including requirements for highly effective age assurance for pornography services and enforcement authority for the national regulator.
Ofcom guidance on highly effective age assurance (8)	Regulatory guidance	Provides technical guidance on acceptable age assurance methods, proportionality principles, and operational safeguards for platform compliance.
Ofcom statement on age assurance and children’s access (9)	Regulatory policy statement	Sets regulatory expectations for preventing children’s access to online pornography and outlines implementation timelines and compliance pathways.
Ofcom enforcement investigations under the Online Safety framework (10)	Regulatory enforcement documentation	Describes enforcement mechanisms, including investigations, compliance notices, and potential sanctions for non-compliant services.
Information Commissioner’s Office Children’s Code (33)	Data protection regulatory framework	Establishes privacy by design and data protection standards for digital services likely to be accessed by children, relevant to age assurance systems.
UK Parliament Online Safety Bill explanatory notes (35)	Legislative background document	Provides policy rationale, legislative intent, and regulatory context for the development of statutory platform duties.
Civil society and digital rights policy analyses, including Open Rights Group and European Digital Rights (11–13)	Civil society policy commentary	Discuss privacy, data protection, and digital exclusion concerns associated with age verification technologies.
Comparative regulatory frameworks, including the European Union Digital Services Act, Australia Online Safety Act, and United States Children’s Online Privacy Protection Act (22–24)	Comparative policy sources	Provide a contextual comparison of regulatory approaches to protecting minors online across major jurisdictions.
Media and platform reporting on early implementation indicators, including traffic changes and VPN uptake (37–41)	Secondary implementation reporting	Provide early descriptive signals of behavioural and platform responses following implementation of age assurance measures.

Evidence was interpreted according to its relative evidentiary weight. Statutory legislation and regulator documentation were treated as primary policy sources, peer-reviewed studies provided contextual interpretation, and media reporting and platform analytics were treated as lower-certainty implementation indicators rather than evidence of causal policy effects.

The analysis applied the Health Policy Triangle framework to organise the interpretation of documentary evidence across four domains: context, actors, process, and policy content (26). Documentary evidence was interpreted within these domains to identify safeguard design features and governance mechanisms shaping the UK age assurance framework. This study, therefore, examines regulatory architecture and policy design rather than estimating behavioural impacts or policy effectiveness.

## UK age-assurance policy through the health policy triangle

### Analytical framework: health policy triangle

The Health Policy Triangle, developed by Walt and Gilson, provides a widely used framework for analysing how health-related policies are shaped by interactions between policy context, actors, process, and policy content (26). Rather than focusing only on legislative provisions, the framework enables examination of the institutional conditions, stakeholder dynamics, and governance processes that influence policy development and implementation (26).

**Figure 1:**
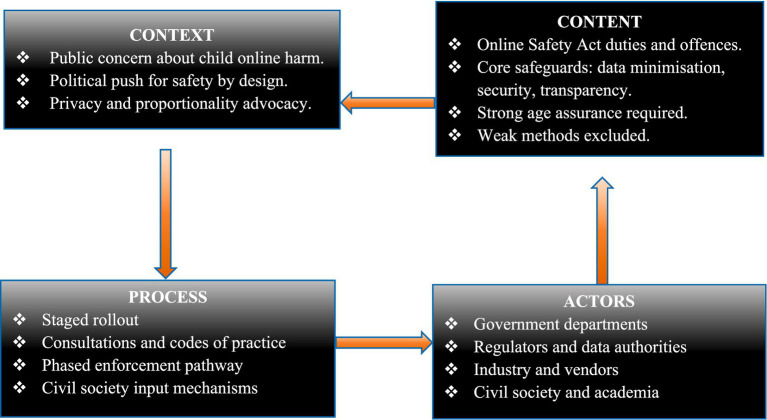
Health policy triangle mapping of the United Kingdom age assurance policy framework. The diagram summarises context, content, process, and actor domains used in this desk-based policy analysis. This diagram is adapted from Walt and Gilson’s health policy triangle framework (26) and UK Online Safety Act and Ofcom regulatory guidance sources (7–10).

In this study, the framework is used to interpret how these four domains structure the UK age assurance policy framework. As illustrated in Figure 1, the triangle highlights how policy context reflects enabling conditions and constraints, policy content captures the design of regulatory safeguards, policy process explains policy formulation and enforcement pathways, and policy actors identify the institutions and stakeholders involved in shaping regulatory outcomes (26, 27).

Taken together, these domains provide a structured basis for examining how the architecture of the UK age assurance framework emerged and how its design features may inform context-sensitive adaptation in SSA. Findings from documentary sources are therefore presented across the four domains of the Health Policy Triangle framework, examining policy context, policy content, policy actors, and policy process in relation to the UK age assurance framework.

### Framework-guided policy analysis of the UK age assurance policy framework

Policy context: Concerns about children’s exposure to sexually explicit material have driven sustained policy debate in the UK, reinforced by evidence on digital harms, screen use, and psychosocial consequences of unfiltered online content (1–4, 31). Regulator data before enforcement of the age assurance policy showed that 8% of children aged 8–14 and 3% of children aged 8–9 in the UK had accessed online pornography in a typical month (32).

These concerns, combined with political commitment to improve online child safety, supported the development of statutory platform duties and regulator-led enforcement, alongside existing data protection law and independent oversight (7, 8).

Civil society organisations, particularly the National Society for the Prevention of Cruelty of Children, strengthened the child protection case by documenting children’s exposure pathways to harmful online content (31). At the same time, privacy and digital rights organisations highlighted the risks related to data collection, user exclusion without formal credentials, surveillance expansion, and algorithmic fairness (11–13). These competing positions shaped the proportionality, privacy, and inclusion safeguards embedded within the regulatory framework.

Policy content: The Online Safety Act 2023 establishes statutory duties of care for search services (for example, general web search engines), user-to-user platforms (such as social media, messaging, and video sharing services where users can post or share content), and dedicated pornography services (7). Key provisions include risk-based child safety requirements, mandatory highly effective age assurance for pornography services, and enforcement powers including fines and access restrictions (7).

Regulatory expectations are further specified in Ofcom guidance, which defines operational safeguards including data minimisation, transparency, user redress, and security by design (8–10). The framework permits multiple age assurance approaches rather than a single mandated method, including document verification, account or device confirmation, mobile operator checks, payment indicators, and privacy-preserving age estimation tools (8–10).

The methods considered ineffective include self-declaration and simple debit card checks, while proportionality principles require that assurance strength matches the level of service risk (8–10, 12, 13). Core safeguard requirements across statutory and regulatory sources include minimal data collection, strong security controls, user communication, appeal mechanisms, and provider reporting duties, with Ofcom holding enforcement authority (8–10).

Policy actors: The UK age assurance framework reflects a multi-actor governance structure involving government, regulators, data protection authorities, industry, civil society, and academic contributors. Central government departments led legislative development, while enforcement responsibilities align with broader online safety objectives (7).

Ofcom holds statutory authority to issue codes of practice, set compliance expectations, and conduct investigations. The Information Commissioner’s Office contributes data protection oversight through regulatory guidance, including the Children’s Code on minors’ personal data (8, 33).

Civil society organisations, academic researchers, and industry actors contributed through advocacy efforts, consultation input, technical evidence, and implementation responses (1–4, 11–13, 31, 34–36). Industry participants, including platforms, assurance vendors, app stores, and service providers, influenced feasibility and proportionality considerations through consultation processes and early compliance decisions (8–10, 23–36).

Policy process: The Online Safety Act 2023 emerged from a multi-year policy development process involving White Papers, public consultations, and parliamentary debate before enactment (7, 31). This process illustrates how political child safety objectives were translated into statutory platform duties through iterative consultation with regulators, civil society, and industry stakeholders.

Agenda-setting and formulation included parliamentary debate and consultation documents that defined duties to reduce children’s exposure to online pornography and recognised age assurance measures as appropriate safeguards (7, 10).

Implementation planning has followed a staged regulatory pathway, with Ofcom issuing guidance, draft codes of practice, compliance timelines, and transition periods to support service readiness (8–10).

Enforcement activity to date consists of regulator investigations, compliance notices, and published enforcement frameworks, which represent regulatory action and planned sanctions rather than evaluated outcomes (9, 10). Ofcom has outlined a proportional escalation pathway that begins with information requests and corrective guidance and may progress to financial penalties or service restrictions in cases of persistent non-compliance (9, 10).

### What we are seeing so far: early implementation signals

These early implementation signals primarily relate to the policy process and actor domains of the Health Policy Triangle, reflecting regulatory enforcement activity, platform compliance behaviour, and stakeholder responses (26).

### Platform compliance and behavioural signals

Implementation and behavioural indicators reported in regulator publications, platform disclosures, and media sources provide early descriptive signals of platform responses and user behaviour as age assurance requirements are introduced (8–10), although these indicators do not constitute causal evidence of policy impact. Several major platforms and adult content services have reported adoption of age assurance tools, often through third-party vendors (8–10), indicating alignment with Ofcom staged guidance and statutory implementation timelines. Media and platform analytics sources report changes in UK visits to some pornography services following the implementation of age checks. Traffic declines are commonly reported in the range of approximately 25 to 40%, with some cases indicating an overall decrease of around one-third, based on third-party web measurement data (37–39). These indicators should be interpreted cautiously, as they cannot distinguish between genuine reductions in exposure and displacement effects, including migration to alternative sites, mirror domains, or circumvention tools such as virtual private networks (38–40). Consistent with this interpretation, survey and media sources report increased virtual private network downloads and migration to alternative platforms, with some providers documenting reported increases exceeding 50% in UK VPN sign-ups following the introduction of age checks (39–41). Technical research on censorship circumvention systems further indicates that users can rapidly bypass blocking and filtering controls, suggesting the potential for behavioural adaptation under age-gating conditions rather than demonstrating direct exposure reduction (42).

### User acceptance and public response signals

User response indicators show that post-enforcement public attitude polling also provides early acceptance signals. A nationally representative survey conducted after the introduction of UK age checks reported that approximately 69% of adults supported the new age restriction rules, although only about 24% believed the measures would be effective in practice, and around 26% reported encountering age verification checks during use (43). Similarly, post-implementation polling found that approximately two-thirds of respondents supported age verification requirements, but fewer than half indicated willingness to submit identity credentials online, indicating conditional acceptance shaped by privacy and trust concerns (44).

### Regulatory, governance process signals, and collaboration

Governance and regulatory process indicators show evolving institutional responses. Public reporting and civil society commentary reflect mixed reactions, combining support for stronger child protection with concerns about privacy, data retention, fairness, and potential errors in age estimation decisions (11–13, 31). Enforcement activity under the Online Safety Act began in July 2025 when Ofcom issued information notices and opened investigations into suspected non-compliant pornography services (10). Subsequent updates indicate progression from preliminary inquiries to confirmed enforcement actions.

In February 2026, Ofcom confirmed non-compliance findings against investigated offenders that had failed to implement highly effective age assurance measures required under Section 12 of the Act, imposing financial penalties of £1,350,000 and requiring implementation of compliant age assurance systems (45). Additional sanctions included a £50,000 fine for failure to respond to regulatory information requests, alongside potential daily penalties of £1,000 for continued non-compliance with age assurance duties and £250 per day for failure to provide requested regulatory information (45). Further investigations have also been opened to assess whether other providers accessible to UK users have implemented compliant age assurance systems (45).

Ofcom is also coordinating with international regulators through the Global Online Safety Regulators Network to develop shared principles covering accuracy, fairness, privacy protection, and proportionate enforcement (46), reflecting increasing recognition that online child safety governance requires cross border coordination.

Taken together, these developments represent early governance and implementation signals rather than outcome evidence. Platform compliance actions, traffic indicators, circumvention patterns, and enforcement responses are therefore interpreted as directional indicators rather than demonstrated policy effectiveness. Peer-reviewed evaluation of behavioural outcomes remains limited, and cross-context applicability requires context-specific assessment (47, 48).

## Can the UK model travel? Transferability to sub-Saharan Africa

### Context and system readiness constraints

To interpret how findings from the UK AAPF analysis may translate to SSA, transferability must be assessed against country-level variation in regulatory capacity, identification coverage, infrastructure, and political economy conditions rather than assuming regional uniformity (47, 48). Countries across the region differ substantially in digital governance capacity, telecommunications infrastructure, and identification systems (17–20, 49, 50). Consequently, implementation pathways are likely to vary across national contexts. Countries with stronger regulatory institutions and higher identification coverage may be able to adopt more stringent verification approaches and shorter compliance timelines, whereas those with weaker supervisory capacity or lower identification coverage may prioritise document-free verification pathways, simplified compliance requirements, and longer transition periods (18, 49, 50).

Infrastructure conditions further shape feasible assurance mechanisms. In many markets, mobile subscriptions and SIM registration systems provide broader coverage than formal identification systems (20, 51), suggesting that mobile operator-based confirmation may be more practical than algorithmic age estimation tools requiring higher device capability and bandwidth (20, 51).

This diversity is reflected in broader governance conditions across SSA, which include low-income, middle-income, and institutionally fragile states with markedly different digital policy capacities (49, 50). Regional initiatives such as the African Union Child Online Safety and Empowerment Policy and the Malabo Convention signal growing political commitment to digital governance, although implementation readiness remains uneven across jurisdictions (17, 20). National assessments, including evidence from Ghana, illustrate this mixed landscape by showing policy momentum alongside institutional and technical implementation gaps (52). Taken together, these variations reinforce that the transferability matrix should be interpreted as a modular analytical framework supporting country-specific calibration of regulatory safeguards rather than a uniform regional prescription.

### Identification, access, and usage conditions

Identification coverage remains limited in many settings, with fewer than half of adults holding formal identity credentials in several countries (18). Nigeria illustrates this constraint, with fragmented and incomplete national identification coverage despite recent expansion efforts (18, 53). At the same time, shared device use in homes, schools, and community access points remains common across multiple countries (20, 54), complicating individual-level assurance approaches compared with more individualised device environments.

Empirical studies from SSA contexts further show that children and adolescents frequently access digital content through shared or household devices with uneven parental supervision and mediation, and that access and oversight patterns vary by socio-economic context (21), supporting the need for flexible and multi-route assurance pathways rather than single credential-dependent models.

### Social legitimacy and risk heterogeneity

Social and cultural conditions also differ across the region. Sexuality, adolescent health, and digital safety remain sensitive policy areas in many communities, shaping acceptance, communication, and uptake of child protection measures (55, 56). Policy transfer therefore depends on social legitimacy as well as technical feasibility. Consistent with this, multi-country and secondary analyses across SSA report that associations between digital media exposure and sexual risk behaviours vary across socio-economic and contextual settings (15), indicating heterogeneous vulnerability and exposure pathways and reinforcing the case for proportional and context-responsive safeguard design.

### Adaptable design principles for transfer

Viewed through the Health Policy Triangle lens, five elements of the UK model appear potentially adaptable when calibrated for differences in regulatory capacity and contextual conditions: (1) risk-based proportionality in assurance strength, (2) multiple verification routes including document-free pathways, (3) transparency and vendor accountability requirements, (4) cross-regulator and cross-sector collaboration mechanisms, and (5) staged supervision with proportionate enforcement pathways (7–10). These elements are analysed as features of regulatory architecture and policy design rather than evidence of policy effectiveness. Table 2 summarises these considerations. Therefore, the matrix is intended as an analytical framework to inform country-level adaptation rather than a uniform regional policy prescription across SSA.

**Table 2:** Transferability matrix illustrating how UK age assurance policy design elements may be adapted across different national contexts in sub-Saharan Africa.

HPT domain	UK AAPF features	Transferable element	SSA adaptation requirements	Risk and mitigation
Context	Risk-based proportionality.	Adjust assurance strength to service type and audience risk.	Calibrate requirements to bandwidth, device access, and regulator capacity.	Risk: Unrealistic accuracy targets.
				Mitigation: Use flexible accuracy ranges and simple baseline standards.
	Multiple routes to prove age.	Telco confirmation, device and account signals, payment evidence, estimation.	Rely more on mobile operator systems due to high mobile use; provide document-free pathways.	Risk: Exclusion for adults without ID.
				Mitigation: Use telco data, SMS or USSD, and accessible appeal routes.
Content	Transparency and vendor assurance.	Accreditation, audit trails, plain language privacy notices.	Use simple privacy notices in local languages and phased vendor accreditation.	Risk: Smaller services may struggle.
				Mitigation: Provide technical support and light initial requirements.
	Privacy-preserving estimation.	Algorithmic estimation for higher risk services.	Use lightweight models on low bandwidth; limit estimation to urban areas where devices allow.	Risk: Poor performance in rural areas.
				Mitigation: Supplement with SMS and operator confirmation.
Process	Staged supervision.	Clear timelines, transition periods, and predictable notices.	Provide longer transition periods and flexible compliance windows.	Risk: Limited regulator capacity.
				Mitigation: Use regional working groups for shared guidance.
	Proportionate enforcement.	Notices, fines, and sparing use of blocking.	Apply fines gradually and reserve blocking for persistent refusal.	Risk: Over-blocking.
				Mitigation: Require an independent review before service restriction.
Actors	Cooperation among regulators, platforms, vendors, and civil society.	Internal and international collaboration with multi-stakeholder oversight and reporting mechanisms.	Collaborate with local stakeholders and leverage the African Union and regional economic communities for cross-border coordination.	Risk: Circumvention and hosting outside jurisdiction.
				Mitigation: Build regional agreements to reduce movement to the least regulated space.

Adapted from the Health Policy Triangle (26) and UK Online Safety materials (Act, Ofcom/DSIT guidance) (7–10, 46), with regional context from African Union policy, GSMA, and World Bank sources (17–20, 51, 55).

### Adapting for equity: practical considerations for sub-Saharan Africa

Adapting the UK age assurance design elements for SSA contexts requires equity-focused calibration of assurance strength levels, verification routes, accountability safeguards, and supervisory pathways, and cross-regulator and multi-stakeholder collaboration mechanisms under varying capacity and infrastructure conditions.

### Proportional assurance and verification pathways

A risk-based model is suitable for heterogeneous regulatory and infrastructure environments, because it allows assurance strength to vary by service risk and local capacity rather than fixed accuracy targets that may be infeasible where connectivity, device quality, and regulator resources differ substantially (17–19, 57). Evidence from regional digital regulation studies shows that flexible compliance models are more implementable where supervisory capacity is limited (54).

Multiple verification routes remain important because identification coverage is incomplete in many countries (18). Since mobile access dominates internet use across the region, regional telecommunications data show that mobile subscriptions and SIM registration systems provide broader coverage than formal identity systems in several markets, making telco confirmation, device signals, and payment proxies more feasible than document-dependent checks in many contexts (18, 20). Shared device environments further require policies that do not assume single-user ownership (20). Privacy-preserving estimation tools may be feasible primarily in urban and higher connectivity settings, consistent with regional infrastructure and device capability evidence (20, 51, 58, 59). Low-connectivity areas are more likely to require operator confirmation, SMS validation, or network-based attestations, which align with existing mobile ecosystems (17, 20). Empirical studies of age estimation systems show performance variability by image quality and demographic factors (60, 61), supporting proportional and context-sensitive deployment rather than universal accuracy thresholds.

### Accountability, compliance burden, and market feasibility

Transparency and vendor accountability provisions align with expanding data protection reforms across several African jurisdictions (62). Regulatory theory and implementation frameworks indicate that simplified and phased compliance models can support uptake among smaller service providers (58). Basic auditability and plain-language privacy notices in local languages support accountability while limiting administrative burden (29, 33).

Cost and market structure also influence feasibility. Platform compliance cost studies and regional digital market analyses indicate that small domestic services face higher proportional compliance burdens (57, 63), supporting phased timelines, proportional fees, and staged supervision models (64). Political economy constraints, including regulator funding, staffing, and technical expertise, remain binding feasibility factors in several jurisdictions and support proportional rather than uniform enforcement expectations (17, 19).

### Collaboration, circumvention, and social acceptability

Circumvention remains a predictable challenge. Studies and reports from multiple jurisdictions document virtual private network use, mirror sites, and offshore migration in response to access restrictions (39–42). Effective mitigation, therefore, depends not only on national supervision but also on cross-border and multi-stakeholder collaboration (46). Regional regulatory coordination evidence suggests that aligned supervisory approaches reduce enforcement gaps (17, 19, 20, 54, 59), supporting the need for collaboration through regional economic communities such as ECOWAS, SADC, and the East African Community.

Implementation effectiveness also depends on language, social norms, and institutional trust. Regional child protection and adolescent health studies indicate higher uptake where digital safeguards are accompanied by community engagement, educator involvement, and parental communication (55, 56, 65). Whole system approaches that involve educators, families, civil society, and industry actors further improve social acceptance and reduce stigma risks (65).

Adaptation across SSA therefore requires context-specific calibration rather than uniform replication (47, 48), and alignment with SDG 3, SDG 10, and SDG 17 objectives, as well as United Nations child rights standards that require online protection measures to balance safety, privacy, and access to information (66, 67).

### Policy implications for sub-Saharan Africa

Drawing on the five adaptable design elements identified and the diversity of SSA regulatory contexts, the following operational policy implications are proposed for national regulators, regional bodies, and research institutions. Actions are separated into near-term implementation priorities and longer-term system development measures to support practical uptake.

### Short-term regulatory and implementation actions

Law and independent oversight: A child rights-centred and risk-based approach to statutory duties should be adopted, defining minimum safeguards, including data minimisation, transparency, and security by design. Furthermore, enforcement authority should be assigned to an operationally independent regulator. In practice, staged enforcement can begin with mandatory risk assessments and improvement notices, followed by corrective action deadlines, before financial penalties or access restrictions are applied in cases of repeated non-compliance.

Multiple assurance routes: Document-free and privacy-preserving assurance pathways should be prioritised alongside stronger verification for high-risk services. Regulators can operationalise telco-based assurance by allowing mobile network operators to issue age status tokens or confirmation flags to platforms without sharing identity data, supported by device or account signals and payment indicators where available. Rapid and low-cost appeal channels should be mandatory.

Equity safeguards: Compliance rules should be designed for low-connectivity and shared device environments by permitting offline or community-based verification points through schools, health centres, or accredited service offices where appropriate. Explicit legal limits should prohibit secondary data use, profiling, or access by law enforcement without due process.

Communication and user support: Providers should be required to publish plain-language privacy notices and child safety explanations in major local languages. National regulators can support implementation through school-based digital literacy programmes and regulator-managed complaint portals with defined response timelines.

### Medium to long-term system development actions

*Regional coordination*: Regional bodies, including ECOWAS, SADC, and the East African Community, can develop baseline assurance standards, mutual recognition frameworks for age status attestations, and shared enforcement protocols to reduce cross-border evasion and compliance costs for smaller providers.

Vendor and technical assurance capacity: Phased vendor accreditation schemes and shared testing resources should be developed to allow smaller domestic platforms to access approved assurance tools without high upfront costs. Regional technical hubs or regulator consortia can host shared audit and validation services.

Cultural and social alignment: Age assurance implementation should be integrated into community engagement strategies that involve educators, parents, youth groups, and civil society organisations to improve trust and uptake. Evidence shows that co-designed communication improves acceptance and compliance (65).

Research priorities in low- and middle-income settings: Longitudinal and mixed-method studies on regional acceptability, behavioural responses, exclusion risks, and child protection outcomes related to age assurance measures should be encouraged. Priority topics include interaction with digital inequality, gender differences in access, and long-term effects on online behaviour and safety.

## Limitations

This study relies on publicly available secondary sources and early implementation signals. No primary data were collected, and no stakeholder interviews or field-based observations were conducted. Therefore, this study does not estimate causal effects or behavioural outcomes. The analysis is based on desk-based policy documents and regulatory source review rather than empirical programme evaluation. Media and industry reports require cautious interpretation because they may reflect selective reporting, measurement limits, and platform self-disclosure bias (30, 68).

This study provides a structured policy analytic framework for assessing design and transferability of age assurance safeguards, not an effectiveness or impact evaluation of the UK model. Future work should include longitudinal and mixed-methods evaluation, stakeholder-based research, and cross-country comparisons to test transferability.

## Conclusion

The UK’s age assurance framework illustrates how regulatory architectures may be structured to support child online protection through risk-based design and multiple verification pathways. This study contributes a policy analytic framework for interpreting and adapting age assurance safeguards across regulatory contexts, based on desk-based documentary analysis rather than outcome evaluation. Translating these design features to the SSA context requires approaches that account for shared device use, limited identification coverage, and socio-cultural diversity while maintaining scalable and privacy-conscious implementation pathways.

Regional coordination and multi-stakeholder collaboration remain important governance considerations for addressing circumvention risks and supporting equitable access to safeguards. Under appropriate regulatory and social conditions, age assurance measures may help strengthen child online protection frameworks while aligning with broader development priorities related to health, inequality reduction, and institutional partnership (SDG 3, SDG 10, SDG 17). Future research should prioritise longitudinal and mixed-method evaluation of age assurance systems, including behavioural responses, exclusion risks, and implementation outcomes across diverse regulatory environments.
